# Detection of transgenes in equine dried blood spots using digital PCR and qPCR for gene doping control

**DOI:** 10.1002/dta.3755

**Published:** 2024-07-11

**Authors:** Jillian Maniego, Caitlin Harding, Jocelyn Habershon‐Butcher, Pamela Hincks, Graham Stewart, Christopher Proudman, Edward Ryder

**Affiliations:** ^1^ Sport and Specialised Analytical Services LGC Fordham UK; ^2^ British Horseracing Authority London UK; ^3^ School of Biosciences and Medicine University of Surrey Guildford UK; ^4^ School of Veterinary Medicine University of Surrey Guildford UK

**Keywords:** digital PCR, dried blood spot, gene doping, qPCR, transgenes

## Abstract

Due to the ease of collection, transport and storage, the use of dried blood spots (DBS) offers an attractive alternative matrix for detection of the abuse of gene therapy, otherwise known as gene doping. This study evaluated the recovery, extraction efficiency and resulting detection capability of DNA from DBS by evaluating different target types, DNA extraction kits, the number of punches and blood tube preservatives. The long‐term storage stability of low‐copy‐number transgene targets in DBS was not assessed in this study but would be noteworthy to investigate further. DNA was quantified using two detection methods: qPCR and digital PCR (dPCR). Using six punches with the Qiagen Investigator kit gave the best overall DNA yield compared with other extraction methods. Including three punches, however, gave better DNA extraction efficiency. Reference material could be detected using qPCR and dPCR in DBS spiked with 5000 copies/mL of blood (approximately 15 copies per 3 mm of punch). The optimal DNA extraction protocol was used on DBS samples from a custom recombinant adeno‐associated virus administration study and showed successful detection of vector targets in DBS samples.

## Graphical abstract

DNA extraction methods for dried blood spots (DBS) were evaluated to determine the suitability of the use of DBS as an alternative matrix for transgene detection towards gene doping screening. DNA was quantified using two detection methods: qPCR and dPCR. Using the Qiagen Investigator kit for DNA extraction with six DBS punches gave a better recovery of targets than three. However, three punches gave better extraction efficiency. DNA can be detected down to 5000 copies/mL of blood using qPCR and dPCR.

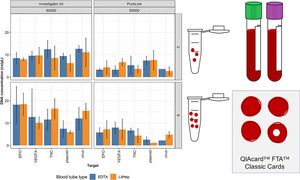

## 1 INTRODUCTION

To ensure fairness in sports and the welfare of athletes and animals, the abuse of gene therapy, otherwise known as gene doping, is prohibited.^1^, ^2^, ^3^ The International Federation of Horseracing Authorities prohibits the use of gene therapy on horses without prior approval from racing authorities. They describe gene therapy as the administration of oligomers or polymers of nucleic acid and genetically modified cells.^2^ Gene doping is described by the World Anti‐Doping Agency (WADA) as the non‐therapeutic use of genes, genetic elements and/or cells that have the capacity to enhance athletic performance.^1^ One example of gene doping is gene transfer, the introduction of exogenous transgenes into living cells,^4^ which could be delivered via a non‐viral vector (i.e., plasmid/naked DNA) or viral vectors (e.g., adeno‐associated virus [AAV]).^5^ In horse racing, guidelines on the ‘Minimum Criteria for Identification of Transgenes’ have been published by the Association of Official Racing Chemists (AORC), and WADA has published guidelines for the detection of transgene doping in whole blood in human athletes using qualitative real‐time PCR (qPCR).^6^

Dried blood spots (DBS) have been used for decades in human diagnostic screening tests^7^ due to their ease of sample collection, transport and storage. There are different types of adsorption matrix that can be used for DBS, with some matrices designed to preserve different analytes such as proteins, steroids and nucleic acids.^8^, ^9^, ^10^, ^11^ Flinders Technology Associates (FTA) classic card matrix is a specialised paper impregnated with chemicals that keeps the DNA stable for long‐term storage at room temperature,^12^, ^13^ which could potentially save cost on freezer storage and will be beneficial for collecting samples, especially when there is no immediate access to a laboratory. Additionally, due to the low blood volume requirement, a skin prick or a small aliquot of blood can be taken without compromising sample volume for other tests.

In routine DBS testing, punched discs with a diameter of 3 or 6 mm are taken.^14^ Various sources have estimated the volume of blood in DBS punches at an average of 3 μL per 3 mm of punch.^11^ Due to the low blood volume, one of the challenges of DBS is dealing with a low‐copy‐number template.^15^ However, DBS have been used in studies such as the detection of foetal DNA in maternal blood^15^ and circulating tumour DNA^16^ where copy numbers are low in circulation blood. Recently, Marchand et al. evaluated the use of DBS as a matrix for gene doping detection.^17^ They reported the detection of 1000 copies of transgenes spiked into a 20‐μL blood spot (50,000 copies/mL of blood). However, levels of transgenes could be lower than this a few days after administration.^18^, ^19^

Heparin has been previously reported to suppress DNA amplification in relation to potassium ethylenediaminetetraacetic acid (K_2_EDTA),^20^ which is widely used to preserve blood for PCR analysis due to DNAse inhibition.^20^, ^21^, ^22^ It was found that there was no significant difference in the quantification of DNA in plasma using qPCR between heparin and K_2_EDTA when blood was processed within 6 h of collection.^21^ Whether heparin will affect the PCR after blood is processed as DBS and stored long term is unknown.

This study aimed to evaluate the use of DBS for transgene detection by qPCR and digital PCR (dPCR). A variety of parameters were tested: two different extraction kits, the number of DBS punches, whole blood collection in K_2_EDTA and lithium heparin (LitHep)‐coated tubes and different transgene carrier types (viral vector, plasmid and double‐stranded synthetic gene blocks).

## 2 METHODS AND MATERIALS

### 2.1 Template construction

Synthetic gene blocks (double‐stranded DNA) containing transgene‐specific assay binding sites for erythropoietin (*EPO*), tenascin‐C (*TNC*) and vascular endothelial growth factor A (*VEGFA*) were designed from the EquCab 3.0 reference genome^23^ and obtained from Thermo Fisher Scientific (Waltham, MA, USA) as lyophilised GeneArt Strings^18^ (Table 1). Lyophilised gene blocks were reconstituted in IDTE pH 8.0 (IDT, Coralville, IA, USA) to a putative concentration of 20 ng/μL before measuring on a Qubit 4 fluorometer (Thermo Fisher Scientific) and diluting into working stocks. Additionally, a custom‐designed plasmid and recombinant AAV vector (designated rAAV8‐CV) containing multiple qPCR assay targets, including *EPO*, *TNC*, *VEGFA* and enhanced green fluorescent protein (*eGFP*), were used. Plasmids were constructed and packaged into AAV8 by VectorBuilder (Chicago, IL, USA). The design of the plasmid and viral vector will be described elsewhere.

**TABLE 1:** Reference material information. The sequence of targets (EPO, TNC and VEGFA) inserted into the plasmid and virus is from the same transcripts as the gene blocks.

DNA type	Target(s)	Length (bp)	Assay design Ensembl transcript ID/GenBank ID
Gene block	EPO	500	ENSECAT00000037087.2
Gene block	TNC	677	ENSECAT00000049833.2
Gene block	VEGFA	569	ENSECAT00000009850.3
Plasmid	EPO, TNC, VEGFA and eGFP	7303	U55761.1 (eGFP)
Virus	EPO, TNC, VEGFA and eGFP	5345	U55761.1 (eGFP)

Abbreviations: eGFP, enhanced green fluorescent protein; EPO, erythropoietin; TNC, tenascin‐C; VEGFA, vascular endothelial growth factor A.

### 2.2 Ethics

Ethical approval was obtained for the administration study. Horses and personnel involved were licensed under the UK Home Office regulations, UK Animals (Science Procedures) Act 1986.

### 2.3 Blood collection

Blank whole blood samples intended for transgene spiking were collected in both 10 mL of K_2_EDTA (Greiner Bio‐One, Kremsmünster, Austria) and 8 mL of LitHep‐based BD Vacutainer™ Eclipse™ PST™ II Plasma Separator tubes (BD, Franklin Lakes, NJ, USA) from one Thoroughbred horse (mare, 5 years old, not used in the transgene administration study) and stored at 4°C for 2 days (due to the timing of collection and other existing laboratory activities) before spiking and spotting.

### 2.4 Administration of custom vector into Thoroughbred horses

The full details of the administration study will be presented in a separate paper. Briefly, two Thoroughbred mares (designated as H1 and H2) received 4.58 × 10^12^ viral particles of the custom vector rAAV8‐CV via intramuscular injection. Whole blood was collected before the administration (0 h) and at defined timepoints over 6 weeks.

### 2.5 Spiking experiment

Resuspended gene blocks, plasmid and rAAV8‐CV virus were initially diluted in 100 ng/μL of poly(A) carrier DNA (Roche, Basel, Switzerland) in LoBind tubes (Eppendorf, Hamburg, Germany) and stored at −20°C. Working concentrations were measured using dPCR before spiking 1 mL of whole blood at 50,000, 20,000, 10,000 and 5000 copies of DNA/mL. Due to the potential variation of blood volumes in each 3‐mm‐diameter hole punch,^11^ the volume of blood used for calculation recovery was defined as 3 μL.^11^ DNA extraction recovery was calculated using the equation below:

$$\%\text{recovery}=\begin{array}{l} \frac{\mathrm{DNA}\;\text{concetration}\left(\frac{\text{copy number}}{\mathrm{\mu L}}\right)\times \text{elution volume}\left(\mathrm{\mu L}\right)}{\left(\text{spiking conc}\left(\frac{\text{copy number}}{\mathrm{mL}}\right)/1000\right)\times \text{number of punches}\times \text{blood spot}\;\mathrm{vol}\left(\mathrm{\mu L}\right)}\\ \times 100. \end{array}$$

### 2.6 DBS preparation

Whole blood was spotted on QIAcard™ FTA™ Classic cards (WB120205, Qiagen, Hilden, Germany) four times at a volume of 125 μL per blood spot. Cards were dried at room temperature in an isolated fume cupboard overnight (or at least 4 h) and then placed in a plastic bag with desiccant at room temperature until processing. Punches were taken from as near the centre of the blood spot as possible using a 3‐mm‐diameter Uni‐Core puncher (Qiagen). After each use, the punching mat and the puncher were cleaned with isopropanol wipes and ethanol.

### 2.7 DNA extraction

DNA was extracted using either the QIAmp Investigator kit (Qiagen) or the PureLink™ Genomic DNA Mini kit (Thermo Fisher Scientific) according to the manufacturer's protocols and eluted at 30 μL. The recommended number of punches for the Investigator kit was 3 × 3 mm in size, and for the PureLink, it was 2–5 punches × 2–3 mm in size. In this study, three and six punches were taken per sample for each kit type for comparative purposes.

### 2.8 qPCR

TaqMan™ assays were designed to target transgenes for *EPO*, *VEGFA*, *TNC* and *eGFP* using the web design tool from Thermo Fisher Scientific or ordered as pre‐designed gene expression assays. Due to the sensitivity of the project towards future gene doping and transgene screening detection, sequences of the assays are not provided. The qPCR assays were tested for efficiency, linearity and specificity using a log_10_ dilution standard curve, equine genomic DNA and no template controls. Only the assays that passed the acceptable criteria^24^ were used in this study. The identity of the qPCR amplicons was verified by adapter ligation and sequencing, as outlined in a previous study.^25^ Assay specificity was determined by a lack of amplification in the presence of blank equine genomic DNA extracted from plasma or whole blood. Results for the PCR efficiency and *R* ^2^ values of the assays used in this study are shown in Supporting Information Data ST1. Samples and controls were performed in triplicate and quantified against a log_10_ dilution standard curve of the appropriate template. Real‐time qPCR reactions were performed on a Viia7 (Thermo Fisher Scientific) using a 384‐well block with the ‘standard curve’ and ‘fast’ modules. A final concentration of 1× of TaqMan™ premixed probe mix was used with TaqMan™ Fast Advanced Master Mix in a 10‐μL final reaction volume containing 1 μL of extracted DNA. Reactions underwent 40 cycles of 95°C for 1 s and 60°C for 20 s. Data were analysed using Quant Studio™ Real‐Time PCR software v1.6.1 (Thermo Fisher Scientific). The quantity of rAAV8‐CV virus was measured using the plasmid standard curve for qPCR. Therefore, the quantity measured was multiplied by 2.^26^ The data were collated in Microsoft Excel and visualised using R. The version number and packages are stated in Data S1. DNA concentrations were expressed as copy numbers per μL (copies/μL).

### 2.9 dPCR

dPCR reactions were performed on the Qiagen QIAcuity using a 24‐well 26K nanoplate. A final concentration of 1× of TaqMan™ premixed probe mix was used with an QIAcuity Probe PCR Kit (Qiagen) in a final reaction volume of 40 μL containing 3 μL of extracted DNA. Reactions underwent 2 min at 95°C, followed by 40 cycles of 95°C for 15 s and 60°C for 30 s. The data were collated in Microsoft Excel and visualised using R. The version number and packages are stated in Supporting Information Data SP1.

## 3 RESULTS AND DISCUSSIONS

### 3.1 Extraction conditions

#### 3.1.1 Extraction kit comparison

**FIGURE 1:**
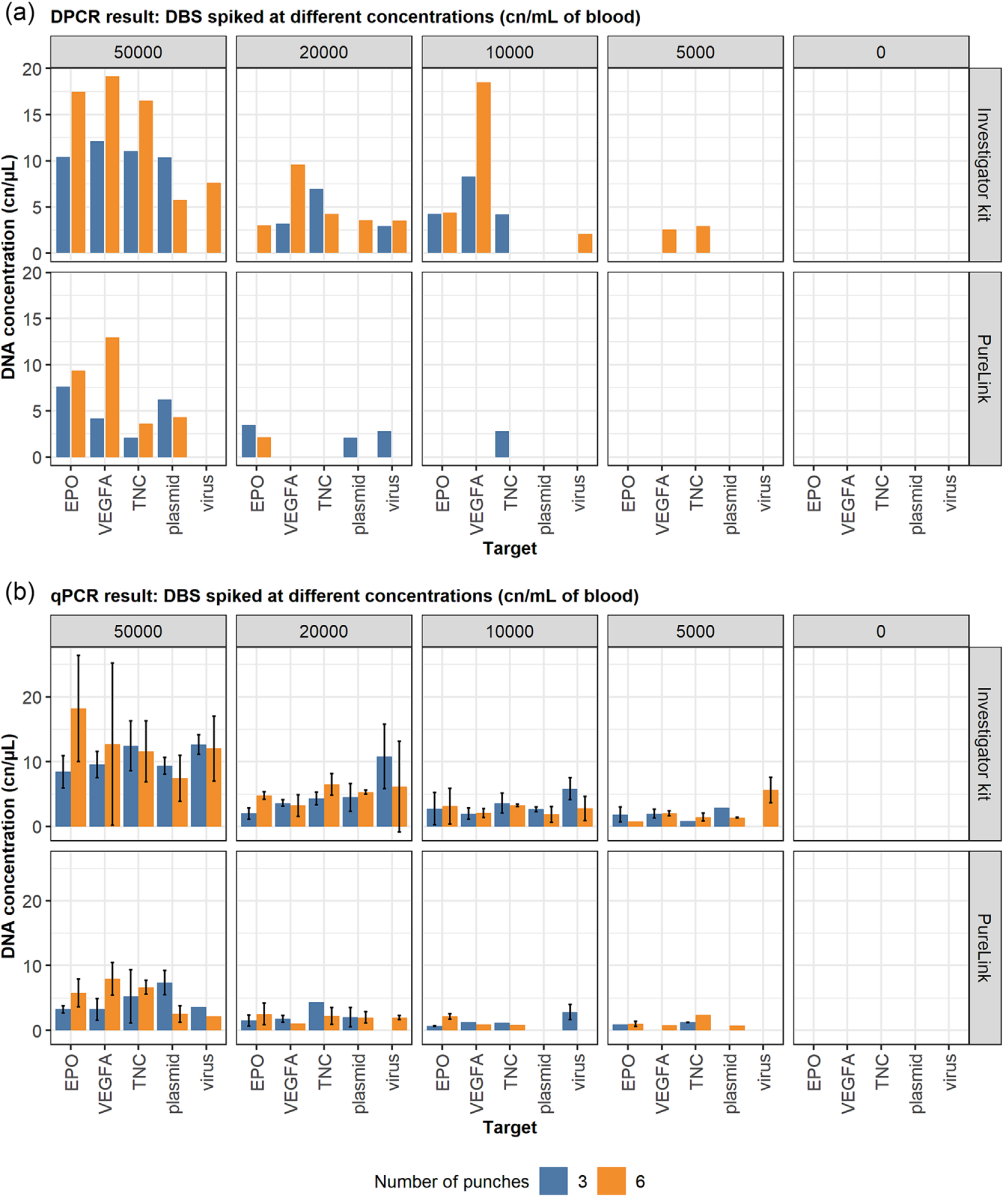
(a) Digital PCR (dPCR) results showing DNA concentration (copies/μL) for dried blood spots (DBS) from K_2_EDTA‐preserved blood extracted using two different extraction kits, 3 or 6 × 3 mm of DBS punches, and spiked at different concentrations of gene blocks, rAAV8‐CV plasmid or rAAV8‐CV virus. (b) qPCR results for the DBS samples. Error bars represent the mean DNA concentration ± standard deviation. Plasmid and virus samples were amplified using the green fluorescent protein assay. EPO, erythropoietin; TNC, tenascin‐C; VEGFA, vascular endothelial growth factor A.

Whole blood was spiked with different types of reference material: linear double‐stranded DNA gene blocks, circular plasmid DNA or single‐stranded rAAV viral DNA at varying concentrations. DNA was extracted with either the QIAmp Investigator kit or PureLink. The Investigator kit is one of the most commonly used kits to reliably extract DNA from FTA cards.^27^ PureLink was chosen for its advertised rapid protocol (2‐h protocol with fewer steps) at a lower cost and was used by Marchand et al. to extract DNA from DBS for transgene detection.^17^ Results are shown in Figure 1a,b. Overall, using the Investigator kit recovered a higher DNA concentration by both qPCR and dPCR, and a better recovery of all the target types compared with PureLink (Supporting Information Data SF1). The Investigator kit also recovered all the targets at lower spiking concentrations by qPCR compared with the PureLink kit.

#### 3.1.2 Number of punches

Generally, six punches gave a higher overall level of DNA for all target types (Figure 1). This was expected because more punches equated to a higher volume of blood. Including six punches in the extraction, in theory, should double the amount of blood in each extraction and therefore recover double the amount of DNA. However, this was not the case using either extraction kit (Supporting Information Data SF1), and including three punches resulted in a more efficient extraction per volume compared with using six.

A subset of samples gave DNA recovery that exceeded 100%, which was unexpected, especially samples spiked at 5000 and 10,000 copies of reference material. The reason for this is unclear but may be due to the uneven distribution of blood, low copies of target molecules on the card and the volume of the literature used in the calculation,^11^ which may bias the result.

#### 3.1.3 Blood tube type comparison

LitHep and K_2_EDTA blood tube types were used to investigate their suitability for blood collection for transgene detection in DBS.

**FIGURE 2:**
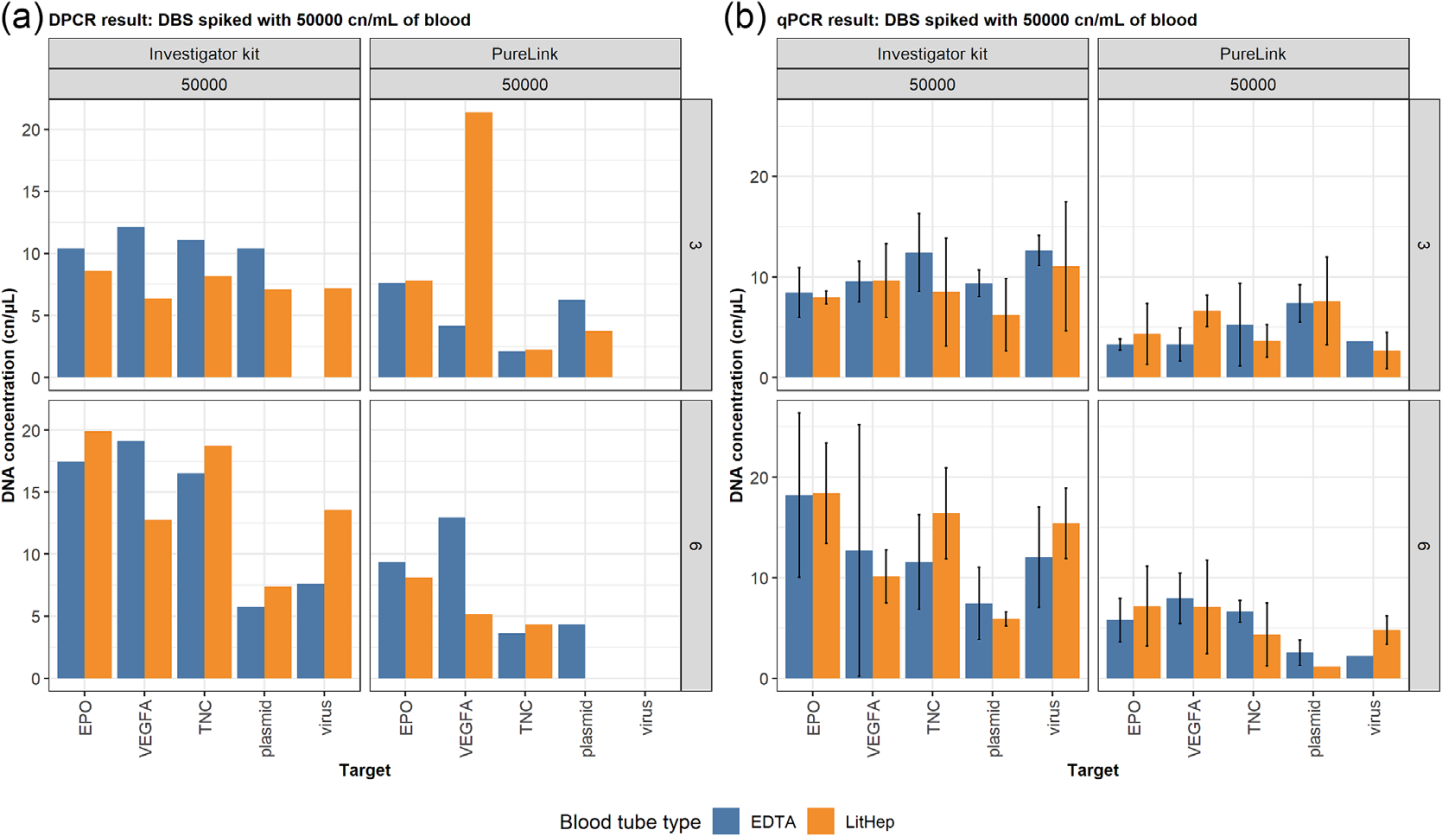
Comparison of DNA copy numbers recovered from dried blood spots (DBS) samples from lithium heparin (LitHep) and K_2_EDTA‐preserved blood spiked with 50,000 copies/mL. DNA extraction was performed using the Investigator kit and with 3 or 6 × 3 mm of DBS punches. (a) Digital PCR (dPCR) results; (b) qPCR results. Error bars represent the mean DNA concentration ± standard deviation. Plasmid and virus samples were amplified using the green fluorescent protein assay. EPO, erythropoietin; TNC, tenascin‐C; VEGFA, vascular endothelial growth factor A.

An example of whole blood spiked with 50,000 copies of DNA per target per 1 mL is shown in Figure 2. Full data are shown in Supporting Information Data SF2. DNA copies recovered from LitHep and K_2_EDTA‐preserved blood samples were comparable across all input concentrations and DNA types, highlighting the suitability of both blood tube types for the blood collection for DBS towards transgene detection.

#### 3.1.4 Blanks

Blank DBS samples were prepared as previously using non‐spiked whole blood from either blood tube and extracted using two different kits with either 3 or 6 × 3 mm of punches. It was a challenge to fully eliminate spurious amplification in all 26 k partitions using dPCR. In this study, blanks and non‐template controls (NTCs) showed positive signals (around 20% of the blanks) on up to two partitions. Therefore, samples with two or fewer positive partitions were considered negative by dPCR.

Initially, amplification of *eGFP* was observed in a subset of blanks with three and six punches by both qPCR and dPCR, suggesting that the source was carried over from the plasmid or viral rAAV8‐CV vectors. Re‐extraction of the blank samples showed no amplification of any targets by qPCR or over the thresholds set for dPCR, suggesting that DNA was carried over from the extraction step. This highlights the importance of cleaning the puncher thoroughly after each use to avoid the risk of false positives. For the screening routine, any putative positive results observed in any samples will be subjected to re‐extraction and confirmatory or sample B analysis.

### 3.2 Comparison of spiking experiment detection techniques

Samples were performed in triplicate reactions on qPCR. In this study, detection in 2/3 or 3/3 reactions was considered a positive finding, and 1/3 or 0/3 a negative finding (Supporting Information Data T2). The percentage recovery for the full data set is shown in Supporting Information Data SF1.

The lowest concentration where all the targets were detected using qPCR was 10,000 copies/mL of blood of any blood tube type using the Investigator kit. By dPCR (Supporting Information Data SF2), the lowest concentration where all targets were reliably detected above the threshold in K_2_EDTA DBS was 20,000 copies/mL of blood using six punches and the Investigator kit, and 10,000 copies/mL using three or six punches and the Investigator kit for LitHep. The lowest concentration at which any target was detected was 5000 copies/mL of blood, or approximately 15 copies per punch by both detection methods.

### 3.3 Comparison of DBS numbers of punches in administration samples

**FIGURE 3:**
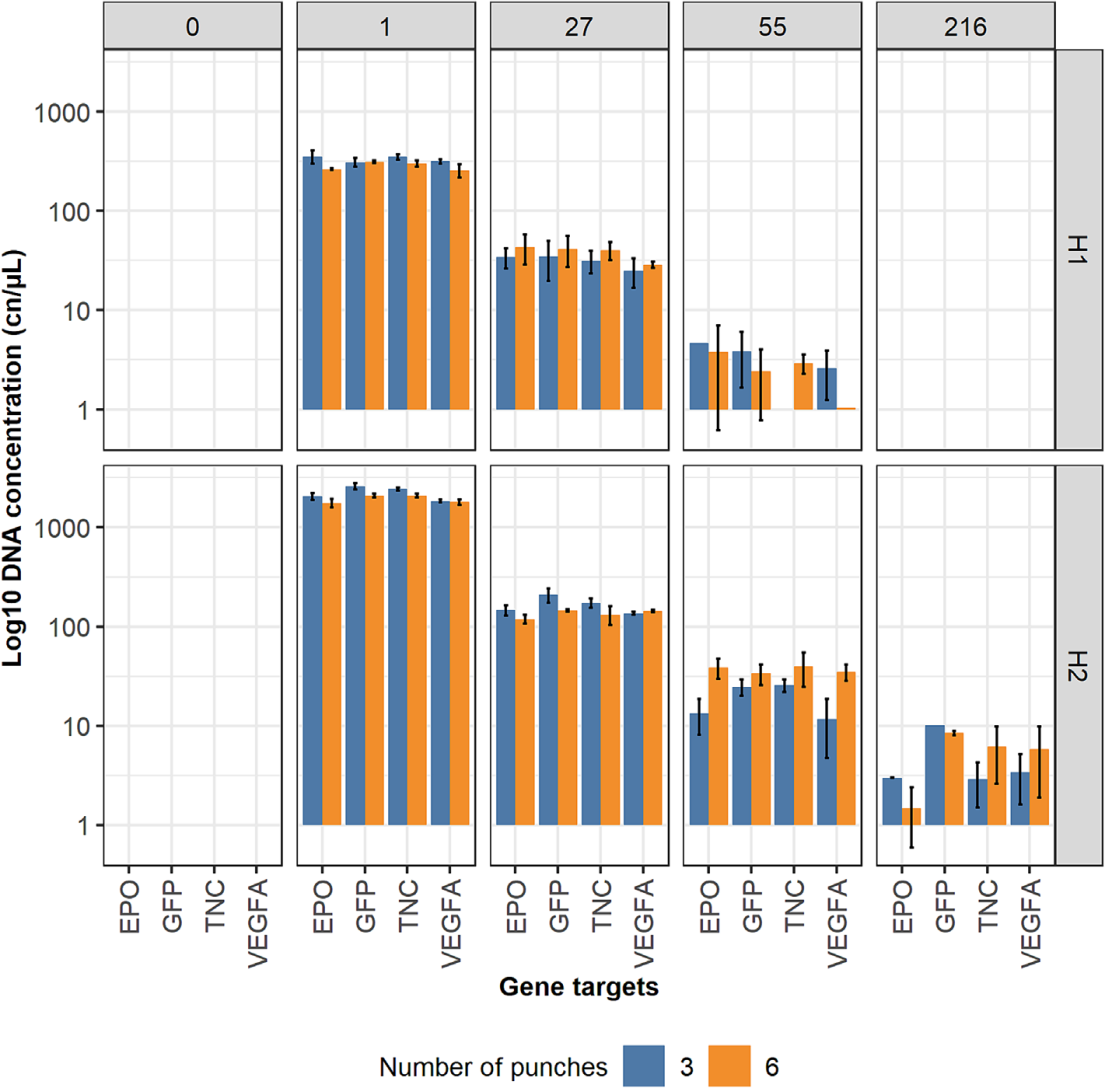
Concentration of DNA samples extracted from 3 × 3 mm of dried blood spots (DBS) and 6 × 3 mm of DBS for two horses between 0 and 216 h post‐administration. Note that the y‐axis scale is adjusted to the log_10_ scale for visualisation. EPO, erythropoietin; GFP, green fluorescent protein; TNC, tenascin‐C; VEGFA, vascular endothelial growth factor A.

The protocol for the Investigator kit was tested on samples from the rAAV8‐CV administration study. DNA from blood samples collected in K_2_EDTA tubes and spotted as DBS was extracted using the Investigator kit with three or six punches. qPCR was performed on four representative (high and low concentration) timepoints from horse H1, and five representative timepoints for horse H2 (Figure 3). The full timepoint dataset with three punches will be published elsewhere as part of the wider administration study.

When extracting with three punches, the last timepoint at which all four targets were amplified in 2/3 or more reactions was at 27 and 55 h post‐administration for horses H1 and H2, respectively. Increasing the number of punches to six did not double the amount of DNA recovered. However, recovery of additional targets at later timepoints was more successful with six punches compared with three punches, shifting the detection time in horse H2 to 216 h (Table 2).

**TABLE 2:** qPCR was performed in triplicate on the dried blood spots from two horses (H1 and H2) following the administration of a customised adeno‐associated virus vector. For this study, 2/3 or 3/3 reactions showing amplification were considered positive, and 1/3 or 0/3 reactions showing amplification were considered negative.

			Gene			
Horse	Punches	Hours	EPO	eGFP	TNC	VEGFA
H1	3	0	Neg	Neg	Neg	Neg
		1	Pos	Pos	Pos	Pos
		27	Pos	Pos	Pos	Pos
		55	Neg	Pos	Neg	Pos
	6	0	Neg	Neg	Neg	Neg
		1	Pos	Pos	Pos	Pos
		27	Pos	Pos	Pos	Pos
		55	Pos	Pos	Pos	Neg
H2	3	0	Neg	Neg	Neg	Neg
		1	Pos	Pos	Pos	Pos
		27	Pos	Pos	Pos	Pos
		55	Pos	Pos	Pos	Pos
		216	Pos	Neg	Pos	Pos
	6	0	Neg	Neg	Neg	Neg
		1	Pos	Pos	Pos	Pos
		27	Pos	Pos	Pos	Pos
		55	Pos	Pos	Pos	Pos
		216	Pos	Pos	Pos	Pos

Abbreviations: eGFP, enhanced green fluorescent protein; EPO, erythropoietin; Neg, negative; Pos, positive; TNC, tenascin‐C; VEGFA, vascular endothelial growth factor A.

Increasing the number of punches did not improve the recovery of targets or the DNA concentration recovered at the last timepoint when analysed by dPCR.

## 4 CONCLUSION

This study has investigated the detection of transgenes from DBS by spiking blood with different concentrations of different DNA fragment sizes and types (synthetic linear double‐stranded gene blocks, plasmids and viruses) and extracting three or six punches using two extraction kits. The optimal extraction protocol to extract the highest yield of transgenes from DBS was using the Qiagen Investigator kit with six punches. However, using three punches gave a better recovery per μL of blood. Results revealed that K_2_EDTA and LitHep anticoagulants were both suitable for blood collections for DBS transgene detection. The study has demonstrated the successful detection of transgene DNA from DBS down to 5000 copies/mL of blood, equivalent to 15 copies per 3 mm of punch, using qPCR and dPCR. The application of the Qiagen Investigator kit on post‐administration DBS samples was successful, and the vector was detected at low levels. Increasing the punches to six did not result in a higher yield at the timepoints tested but did lead to the successful detection of more targets in qPCR replicates. This further highlights the potential capabilities of DBS as an alternative matrix for gene doping detection.

Nucleic acid has been reported to be stable in FTA cards when stored long term at room or ambient temperature.^12^, ^13^ Whether this is true for low‐copy‐number DNA transgenes would be noteworthy to investigate further. The investigation of the stability of transgene in FTA cards after long‐term storage was not explored, as it falls outside the original remit of this study. However, long‐term storage would be noteworthy to explore in the future and would be useful to provide guidance on storage conditions for FTA cards for gene doping detection and would be useful for potential retrospective analysis of transgenes. Future work could also include using blood from a larger population, using different absorbent matrixes (i.e., VAMS™),^17^ spotting FTA cards with different volumes of blood and fixing different matrixes on cards (i.e., plasma and urine).

## CONFLICT OF INTEREST STATEMENT

The authors declare no conflict of interest.

## Supporting information


**Data S1.** Supporting Information

## Data Availability

Restrictions apply to the availability of these data, which were used under license for this study. Data are available from the authors with the permission of the British Horse Racing Authority.
