# Supplementary material for: Detection of transgenes in equine dried blood spots using digital PCR and qPCR for gene doping control
Source: Drug Test Anal. 2024 Jul 11;17(5):626–33. doi: 10.1002/dta.3755 (PMC12012414; doi:10.1002/dta.3755)

Supplementary data

# Supplementary Protocol SP2: R packages

- R version used at the time of experiment: version 4.2.1
- R packages used:

1. utils_4.2.1
2. dplyr_1.1.0
3. ggplot2_3.4.1
4. ggthemes_4.2.4

## ST 1.

qPCR assay efficiency and R2 (linearity) values for gene targets and different assay types. PCR efficiencies between 90% and 110%, and R2 values >0.980 were considered acceptable.

| Gene | Type | PCR efficiency | R-squared value |
| --- | --- | --- | --- |
| EPO | TaqMan | 100.118 | 1 |
| TNC | TaqMan | 102.594 | 0.995 |
| VEGFA | TaqMan | 101.711 | 0.996 |
| EGFP | TaqMan | 100.256 | 0.998 |

## ST 2.

Spiked DBS samples were performed on QPCR in triplicate reactions. For this study, 2/3 or 3/3 reactions showing amplification were considered as positive and 1/3 or 0/3 reactions showing amplification were considered as negative. Positives are highlighted in green to aid visualisation.

| Extraction Kit | Tube type | Target | 3 | | | | 6 | | | |
| --- | --- | --- | --- | --- | --- | --- | --- | --- | --- | --- |
|  |  |  | **5000** | **10000** | **20000** | **50000** | **5000** | **10000** | **20000** | **50000** |
| Investigator Kit | EDTA | assay AAV | Neg | Pos | Pos | Pos | Pos | Pos | Pos | Pos |
|  |  | assay plasmid | Neg | Pos | Pos | Pos | Pos | Pos | Pos | Pos |
|  |  | EPO | Pos | Pos | Pos | Pos | Neg | Pos | Pos | Pos |
|  |  | TNC | Neg | Pos | Pos | Pos | Pos | Pos | Pos | Pos |
|  |  | VEGFA | Pos | Pos | Pos | Pos | Pos | Pos | Pos | Pos |
|  | LitHep | assay AAV | Neg | Pos | Pos | Pos | Neg | Pos | Pos | Pos |
|  |  | assay plasmid | Pos | Pos | Pos | Pos | Neg | Pos | Pos | Pos |
|  |  | EPO | Neg | Pos | Pos | Pos | Neg | Pos | Pos | Pos |
|  |  | TNC | Pos | Pos | Pos | Pos | Pos | Pos | Pos | Pos |
|  |  | VEGFA | Pos | Pos | Pos | Pos | Pos | Pos | Pos | Pos |
| PureLink | EDTA | assay AAV | Neg | Pos | Neg | Neg | Neg | Neg | Pos | Neg |
|  |  | assay plasmid | Neg | Neg | Pos | Pos | Neg | Neg | Pos | Pos |
|  |  | EPO | Neg | Pos | Pos | Pos | Pos | Pos | Pos | Pos |
|  |  | TNC | Pos | Neg | Neg | Pos | Neg | Neg | Pos | Pos |
|  |  | VEGFA | Neg | Neg | Pos | Pos | Neg | Neg | Neg | Pos |
|  | LitHep | assay AAV | Neg | Pos | Neg | Pos | Neg | Neg | Neg | Pos |
|  |  | assay plasmid | Pos | Neg | Pos | Pos | Neg | Pos | Neg | Neg |
|  |  | EPO | Pos | Pos | Neg | Pos | Pos | Pos | Neg | Pos |
|  |  | TNC | Neg | Pos | Pos | Pos | Pos | Pos | Neg | Pos |
|  |  | VEGFA | Neg | Pos | Pos | Pos | Pos | Pos | Pos | Pos |

## SF 1.

A) DPCR results showing DNA percentage recovery (%) of DBS using 2 different extraction kits, 3 and 6 punches and different spiking concentrations of gene blocks, plasmid, or virus. B) qPCR results for the DBS samples. Error bars represent the mean DNA concentration ± standard deviation.


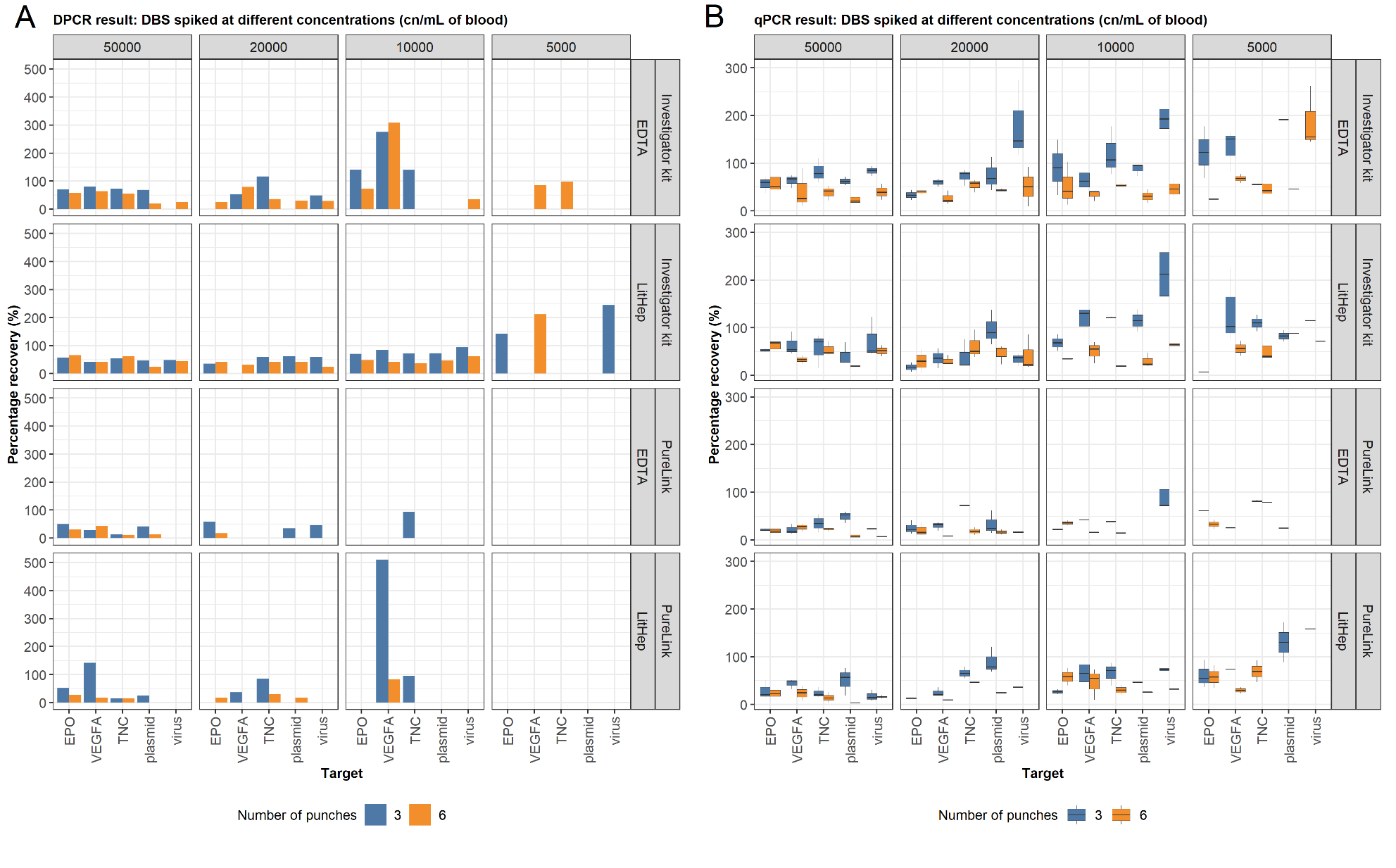


## SF 2.

DPCR results for DBS samples spiked with varying concentrations of reference material and extracted using Investigator kit or PureLink with 3 or 6 DBS punches. Samples with 2 or less positive partitions were considered a negative and therefore taken out of the data set.


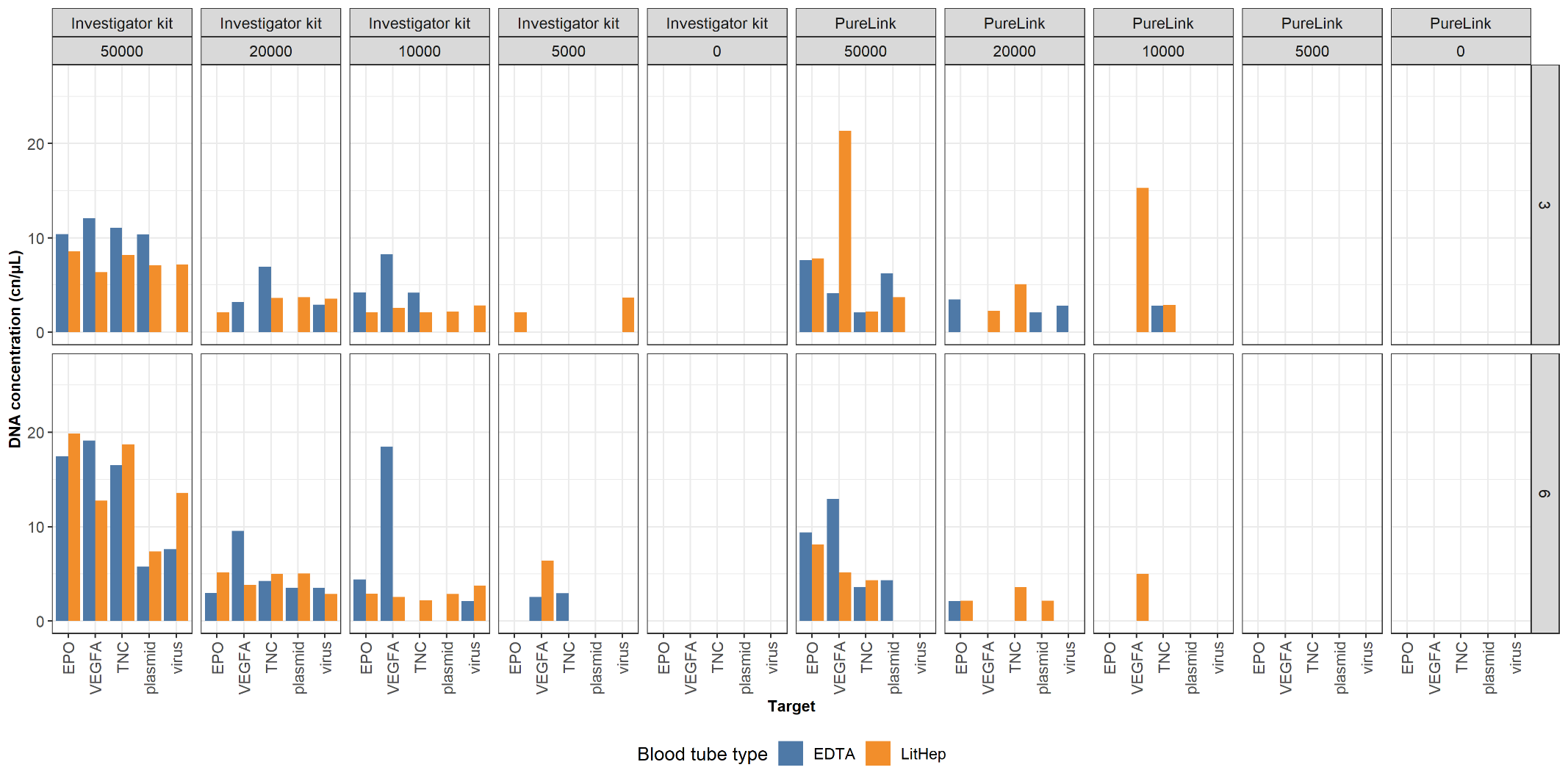

Supplement: Supplementary file 1 — Data S1. Supporting Information [file DTA-17-626-s001.docx]
